# Prevalence and factors associated with non-adherence to multi-drug resistant tuberculosis (MDR-TB) treatment at Mulago National Referral Hospital, Kampala, Uganda

**DOI:** 10.4314/ahs.v21i1.31

**Published:** 2021-03

**Authors:** Charles Batte, Martha S Namusobya, Racheal Kirabo, John Mukisa, Susan Adakun, Achilles Katamba

**Affiliations:** 1 School of Medicine, University of Liverpool; 2 Uganda Tuberculosis Implementation Research Consortium; 3 Lung Institute, Makerere University College of Health Science; 4 Clinical Epidemiology Unit, Makerere University College of Health Sciences

**Keywords:** Non-adherence, multi-drug resistant tuberculosis, treatment

## Abstract

**Background:**

In Uganda, 12% of previously treated TB cases and 1.6% of new cases have MDR-TB and require specialized treatment and care. Adherence is crucial for improving MDR-TB treatment outcomes. There is paucity of information on the extent to which these patients adhere to treatment and what the drivers of non-adherence are.

**Methods:**

We conducted a cohort study using retrospectively collected routine program data for patients treated for MDR-TB between January 2012 – May 2016 at Mulago Hospital. We extracted anonymized data on non-adherence (missing 10% or more of DOT), socio-economic, demographic, and treatment characteristics of the patients. All participants were sensitive to MDR-TB drugs after second line Drug Susceptible Testing (DST) at entry into the study. Factors associated with non-adherence to MDR-TB treatment were determined using generalized linear models for the binomial family with log link and robust standard errors. We considered a p- value less than 0.05 as statistically significant.

**Results:**

The records of 227 MDR- TB patients met the inclusion criteria, 39.4% of whom were female, 32.6% aged between 25 – 34 years, and 54.6% living with HIV/AIDS. About 11.9% of the patients were non-adherent. The main driver for non-adherence was history of previous DR-TB treatment; previously treated DR-TB patients were 3.46 (Adjusted prevalence ratio: 3.46, 95 % CI: 1.68 – 7.14) times more likely to be non-adherent.

**Conclusion:**

One in 10 MDR-TB patients treated at Mulago hospital is non-adherent to treatment. History of previous DRTB treatment was significantly associated with non-adherence in this study. MDR-TB program should strengthen adherence counselling, strengthen DST surveillance, and close monitoring for previously treated DR-TB patients.

## Introduction

Multi Drug Resistant / Rifampicin Resistant TB (MDRTB/RR-TB) is one of the global public health problems. It remains a priority for the World health Organization through the End Tby 2030 strategy^1^. In 2018 alone, 186,772 cases of MDR-TB/RR were detected and notified globally^3^, ^5^. During the same year, Africa registered an estimated MDR-TB/RR-TB incidence rate of 7.3 per 100 000 population^5^. Uganda remains a high-burden country with 12 % of previously treated TB cases and 1 % of new cases having MDR-TB^5^. With the high MDR-TB/RR-TB numbers prompt diagnosis, start, and adherence to treatment by patients is key. MDR-TB treatment is difficult, complex and prolonged (with an approximate duration of 18 to 24 months). It is also expensive with an estimated treatment cost of greater than 40 times that of drug susceptible TB especially in resource limited countries^7^.

Daily Injectable aminoglycosides form the backbone of MDR-TB treatment regimens and their prolonged use is associated with better treatment outcomes^6^. Current recommendations approve the use of aminoglycosides for eight months during the intensive phase of MDRTB treatment^8^. However, aminoglycosides are associated with adverse events that include hearing loss which can be permanent and may also affect the quality of life of patients who may be cured of MDR-TB but face life-long disability^9^. Other adverse events due to MDRTB treatment include; renal failure and nerve damage^10^. These adverse effects combined with the long treatment period and cost to the patients immensely affect patients' adherence to treatment and therefore, treatment outcomes.

Despite TB being a curable disease, ≥ 90% patient adherence to anti-TB treatment is required to achieve it^11^. In Uganda, the burden of non-adherence among patients with drug susceptible TB is high^12^. Non-adherence has been associated with medication drug side effects, length of treatment, pill burden, and the economic impact of accessing TB treatment (especially when directly observed). In a study done at Mulago National Referral Hospital^13^, it was found that 59% of patients with drug susceptible Tuberculosis had poor treatment outcomes such as treatment failure, loss to follow-up and death. No previous study has however, been done among MDR-TB patients. Non-adherence to medication results into treatment failure, TB relapse, and development of multi-drug-resistant TB (MDRTB) and extensively drug-resistant TB (XDR-TB), the emergence of which represents an unprecedented public health emergency^14^.

An understanding of the factors associated with non-adherence to MDR-TB treatment is imperative in generating evidence that can inform and guide development of programs and initiatives to improve adherence patterns, MDR-TB treatment outcomes and ending TB. In this paper, we present the prevalence and factors associated with non-adherence to MDR-TB treatment using secondary data from Mulago National Referral Hospital the largest MDR-TB treatment center in Uganda.

## Methods

### Study Design

We conducted a cohort study using secondary data from patients' records (treatment files) at Mulago National Referral Hospital (MNRH) TB center. The use of secondary data allows for the analysis of large sets of routinely collected data at low cost 15.

### Setting

MNRH TB center registers between 50–70 new MDRTB patients per year under the Uganda National Tuberculosis and Leprosy Program (NTLP). It is one of the 13 MDR-TB treatment centers across the country where new MDR-TB patients are referred and treated. T/span>rained health workers and treatment supporters carry out Directly Observed Therapy (DOT) for the patients. The health worker maintains a drug record chart for each administered dose of the MDRTB drugs and provides adherence counselling to each patient. Clinical data are manually collected using the patients' treatment card and entered into three National registers; the presumptive TB register, the laboratory register, and the treatment register. It includes; the name of the patient, age, sex, HIV status, marital status, date of clinic attendance, date of sputum submission, results of sputum smear, results of the Gene Xpert test, TB diagnosis, type of treatment and treatment regimen. At a periodic basis, all the data from the 13 MDR-TB treatment centers is entered into the NTLP's electronic database hosted at the Ministry of health, while the physical records (back-up hard copies) including MDRTB patient's cards (after completion of treatment) are stored at the MNRH TB center. Before commencing of MDR-TB treatment, all patients are evaluated for sensitivity to current MDR-TB drugs after second line Drug Susceptibility Testing (DST) to rule out X-DR TB. However, due to personnel and logistical challenges, this electronic database was not current at the time of the study. We, therefore, only considered patient files from MNRH TB center for data abstraction in order to meet the objectives of our study.

We estimated the sample size using the Kish Leslie (1965) formula by assuming the prevalence of non-adherence among DR-TB patients 32.7% as reported in South Africa^16^, 254 participants would give the prevalence of non-adherence with a 95% confidence and 5 % margin of error. However, because of incompleteness of routinely collected data, we were only able to collect 227 participant charts for analysis in this study.

### Study population

We considered the records of all MDR-TB patients treated at MNRH TB unit between January 2012 and May 2016. We extracted participants' data from the start to end of their MDR-TB treatment for the period under review. The length of the records included ranged between 18- 24 months (varied according to the prescribed duration of treatment by the attending physician) of treatment. We excluded records of the patients with missing data on key independent variables like age, sex, treatment category, HIV status, previous TB treatment from the final analysis.

### Study procedure

After obtaining ethical clearance from the IRB and permission from the NTLP, the principal investigator accessed the electronic MDR-TB register, abstracted data from the charts where possible, and set up the dataset as informed by the variables under consideration in this study.

### Data quality control

A data extraction questionnaire was pretested on less than 5 % of the sample size and adjustments made before conducting final data collection. Fifteen records were ineligible because of missing data. The excluded and included records had negligible difference in non-adherence levels.

### Data analysis

In preparing for data analysis, we merged all data to create a final data set. Database access was locked on the 15th September 2016 permitting no further edits. The raw data set was imported into STATA version 14.0 for analysis (College station, Texas, USA). We computed means, medians, standard deviations and interquartile ranges for continuous variables; proportions and percentages for categorical variables. Non- adherence was defined as a patient missing ≥ 10 % of the total prescribed dose over the 18–24 months of treatment^14^, ^17^. The prevalence of non- adherence was calculated as a proportion defined as the number of individuals that were non-adherent divided by the total number of participants included in the study.

We evaluated the factors associated with non-adherence to MDR-TB treatment using generalized linear models for the binomial family with a log link and robust standard errors. Factors with p value less than 0.25 and potential confounding variables were considered for multivariable regression analysis. We formed two-way interaction terms between the variables to assess for interaction. The crude and adjusted prevalence ratios and their 95 % confidence intervals were calculated. A p-value of less than 0.05 was considered statistically significant.

### Ethical consideration

The School of Biomedical Sciences – Higher Degrees Research and Ethics Committee (SBS-HDREC #549), approved the study protocol and a waiver of informed consent while Mulago Hospital Research Ethics Committee (MHREC) granted administrative approval. MNRH and NTLP granted final permission to the study team to access the patient records the before final data extraction. We maintained confidentiality of the patients' data by using anonymized extracted data for further analysis.

## Results

### Socio-economic and treatment characteristics of participants

Eighty-nine (34.9%) of the participants were female, seventy-four (32.6%) were aged between 25 and 34 years of age and 80/227 (38.5%) were married. About 65.1% of the participants were employed and one hundred eighteen (52%) lived in rural settings.

One hundred twenty-four (54.6%) of the participants were co-infected with HIV while one hundred fifty-one (66.5%) had had TB treatment before (Table 2).

**Table 2:** Treatment characteristics of study participants with MDR-TB at Mulago National Referral Hospital TB unit, January 2012-May 2016, N=227

Variable	Frequency, N	Percentage %
**HIV Status**		
Negative	103	45.4
Positive	124	54.6
**On ART** *		
No	105	46.3
Yes	122	53.7
**Treatment registration group**		
New (N)	79	34.8
Relapse (Rp)	39	17.2
After loss to follow-up (Rt)	30	13.2
After failure of first treatment with first-line drugs (F1)	41	18.1
After failure of retreatment regimen with first line drugs (F2)	35	15.4
After failure of regimen with second-line drugs (F4)	1	0.4
Other / previously treated without known outcome(o)	2	0.9
Previous TB** Treatment		
No	76^a^	33.5
Yes	151	66.5
Previous DR-TB** Treatment		
No	208	91.6
Yes	19	8.4
**Comorbidities**		
No	87	38.3
Yes	140	61.7
**Adverse Events**		
No	168	74
Yes	59	26
**Month of Sputum Conversion** ****		
0 – 2 months	85	41.5
> 2 months	51	24.9
Unable to produce sputum	69	33.7

* ART: Anti Retro-viral Treatment

** TB: Tuberculosis

*** DR-TB: Drug Resistant Tuberculosis

**** 22 participants missing data

a 3 individuals missing data for this variable

### Non-adherence to MDR-TB treatment

**Figure 1:**
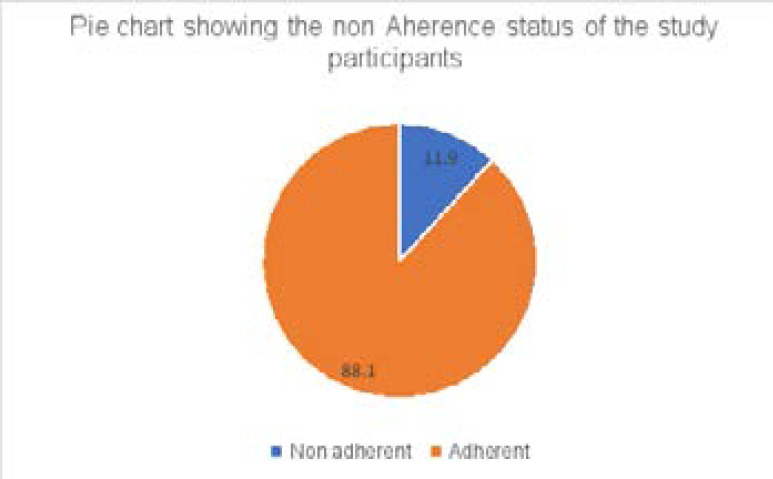


Of the 227 patients with MDR-TB, 27 (11. 9%, 95 %CI: 8.2–16.8) were non-adherent to treatment (Figure 1).

### Factors associated with Non-adherence to MDR-TB treatment

At regression analysis, a previous history of DR-TB treatment and the treatment registration group were significantly associated with non-adherence to treatment (Table 3 and 4).

**Table 3:** Regression analysis for socio-demographic characteristics and association with non-adherence to MDR-TB treatment at Mulago National Referral Hospital TB unit, January 2012–May 2016, N=227

Characteristic	Non adherent, N (%)	Adherent N (%)	Crude prevalence ratios (95% Confidence intervals)	P- value
**Sex** *				
Male	8 (30.8)	81 (40.5)	I	
Female	18 (69.2)	119 (59.5)	1.46 (0.66–3.22)	0.347
**Age group (in** **years)**				
0–14	1 (3.7)	4 (2.0)	1	
15–24	5 (18.5)	57 (28.5)	0.57 (0.06–2.83)	0.361
25–34	13 (48.2)	61 (30.5)	0.88 (0.14–5.45)	0.889
35–44	5 (18.5)	48 (24.0)	0.47 (0.07 –3.30)	0.449
45–54	2 (7.4)	24 (12.0)	0.38 (0.04 –3.49)	0.396
>54	1 (8.7)	6 (3.0)	0.71 (0.06–8.95)	0.794
**Marital Status** **				
Married	9 (34.6)	71 (39.0)	1	
Separated	6 (23.1)	26 (14.3)	1.67 (0.64–4.31)	0.292
Single	10 (38.5)	77(42.3)	1.02 (0.44–2.39)	0.960
Widowed	1 (3.9)	8 (4.4)	0.99(0.14–6.96)	0.990
**Employment** ***				
No	11 (40.7)	65 (34.0)	1	
yes	16 (59.6)	126 (66.0)	0.78 (0.38–1.59)	0.494
**Residence**				
Urban	12 (44.4)	97 (48.5)	1	
Rural	15 (55.6)	103 (51.5)	0.83 (0.33–2.05)	0.678

* one participant missing sex variable

** 19 missing marital status

*** 9 missing employment status

**Table 4:** Regression analysis of treatment characteristics and association with non-adherence to MDR-TB treatment at Mulago National Referral Hospital TB unit January 2012-May 2016, N=227

Characteristic	Non adherent, N (%)	Adherent N (%)	Crude prevalence ratios (95% Confidence intervals)	P-value
**HIV status**				
Negative	10 (37.0)	93 (46.5)	1	
Positive	17 (63.0)	107 (53.5)	1.41 (0.68–2.95)	0.359
**On ART**				
No	10 (37.0)	95 (47.5)	1	
Yes	17 (63.0)	105 (52.5)	1.46 (0.70 –3.06)	0.312
**Treatment** **registration** **group**				
New	9 (33.3)	70 (35.0)	1	
Relapse	2 (7.4)	37 (18.5)	0.45 (0.10 –1.99)	0.293
After loss to follow up	8 (29.6)	22 (11.0)	2.34 (0.99–5.51)	0.052
After failure of first treatment with first line drugs	2 (7.4)	33 (16.5)	0.50 (0.11–2.21)	0.362
After failure of regimen with second line drugs and others	2(7.4)	1 (0.5)	5.85 (2.13–16.1)	0.001***
**Previous TB** **treatment**				
No	8 (29.6)	68 (34.0)	1	
Yes	19 (70.4)	132 (66.0)	1.19 (0.55–2.61)	0.654
**Previous DR-TB** **treatment**				
No	19 (70.4)	189 (94.5)	1	
Yes	8 (29.6)	11(5.5)	4.61 (2.33–9.11)	<0.001*
**Comorbidities**				
No	8 (29.6)	79 (39.5)	1	
Yes	19 (70.4)	121 (60.5)	1.47 (0.67–3.23)	0.330
**Adverse events**				
No	22 (81.5)	146 (73.0)	1	
Yes	5 (18.5)	54 (27.0)	0.65 (0.26–1.64)	0.357
**Month of** **sputum** **conversion**				
0–2 months	9 (39.1)	76 (41.8)	1	
> 2 months	9 (39.1)	46 (25.3)	0.92 (0.33–2.62)	0.885
Unable to produce sputum	9 (39.1)	60 (33.0)	1.23 (0.52–2.94)	0.638

At multivariable regression analysis, patients who were treated previously for DR-TB were 3.46 times (Adjusted prevalence Ratio: 3.46, 95 % CI: 1.68 – 7.14) as likely to be non-adherent compared to those that had not been treated for DR-TB before (Table 5).

**Table 5:** Multivariable regression analysis of factors associated with non-adherence to MDR-TB treatment at Mulago National Referral Hospital TB unit January 2012–May 2016

Characteristic	Adjusted Prevalence Ratios (95% CI)	P- Value
**Previous DR-TB Treatment**		
No	1	
Yes	3.46 (1.68 – 7.14)	0.001
**Treatment registration group**		
New (N)	1	
Relapse (Rp)	0.43 (0.10 – 1.78)	0.245
After loss to follow-up (Rt)	1.77 (0.76– 4.12)	0.186
After failure of first treatment with first-line drugs (F1)	0.86 (0.27 – 2.72)	0.803
After failure of retreatment regimen with first line drugs (F2)	0.44 (0.09 – 2.05)	0.295
After failure of regimen with second-line drugs (F4) and others	1.85(0.57–6.05)	0.306

## Discussion

This study set out with the aim of determining to what extent MDR-TB patients previously treated at MNRH under the national TB program in Uganda adhered to treatment. The results of the study showed that 11.9% of the patients are non-adherent to their treatment. This finding is lower than those of other studies done in different settings that reported non-adherence levels between 18–33%^16^–^21^. The differences in rates of non-adherence may be due to different methodologies and definitions used. The findings in our study may also be due to lack of social support^22^, ^23^, treatment drug side effects, forgetting to take medication, being away from home, missing date of appointment, lack of transportation cost to treatment center, poor communication between patient and healthcare providers, and stock out of medicines.

Participants who had been treated for DR-TB previously were 3.46 times more likely to be non-adherent to MDR-TB treatment. Previous studies have described the role of prior susceptible TB treatment in the development of MDR-TB^24^ but have not described its impact on adherence among those being retreated. It is very likely that the same factors like poor social support that lead patients to default on the first DR-TB treatment episode could also be carried into subsequent retreatment episodes and thus influence their adherence. MDR-TB treatment programs therefore must take extra care when dealing with patients that have been treated for DR-TB before. Adherence programs need to incorporate this consideration. The patients that are potential repetitive defaulters pose an extra risk to the efforts of curbing the TB epidemic. Our study participantsere at risk of developing X-DR during the period under review despite them being sensitive to all second line MDR-TB drugs after DST at the start of the treatment. They may also be more likely to continue spreading the disease in the communities where they live^20^.

## Strength

The strength of the current study is the utilization of programmatic data in assessing non-adherence and associated factors. Unlike in clinical trials where the characteristics of those participants are highly selective, routinely collected data gives us the true picture of what is happening in the real world. The inferences made from this data can be used to develop interventions that are relevant to specific contexts and environments in which the patients live on a daily basis. When further developed and analyzed from the patient's perspective it can be used to inform policy makers on how quality of care can be improved and how patient education tools can be developed to fit patient needs.

## Limitations

This study was unable to assess the impact of factors that are not routinely collected within the National Tuberculosis treatment program. Factors such as the patient's level of education, distance from hospital, smoking and lack of awareness of the treatment duration that have been found to significantly impact on adherence in other studies could not be assessed in this study because data was not available.^4^ The quantitative nature of the study focuses on quantifying adherence and associated factors. It thus does not permit the investigation of qualitative patient factors such as self-motivation, counselling experiences, family and nutritional support, that have been previously found to be key drivers of adhrence to MDR-TB treatment^22^. The exclusion of some records of patients missing information on key independent variables under study may have minimally biased our results. However, MNRH TB unit is the largest and receives patients from across Uganda.

## Conclusion

1 in 10 MDR-TB patients treated at the Mulago National Referral Hospital in Kampala, Uganda is non-adherent to treatment. History of previous DR-TB treatment was associated with non-adherence to TB treatment in our cohort.

## Recommendations

The National MDR-TB treatment program should strengthen adherence counselling, provide consistent second line DST during treatment and close monitoring for patients that have been previously treated for MDR-TB. Secondly, because such patients have been exposed to sub-optimal doses of the treatment regimen, they are very likely to develop Extensively Drug Resistant Tuberculosis, which is more lethal. Therefore, to protect the public, TB treatment programs should focus on promoting adherence to MDR-TB treatment and ensuring strict management for those that have been treated before.

The TB enablers program that is currently being used to defray catastrophic costs for MDR-TB patients should also incorporate aspects of adherence, patients that adhere to treatment should be given extra incentives in order to encourage the non-adherent ones to adhere more.

The National MDR-TB program and the Ministry of health should take up the shorter treatment regimens that have been found to be equally effective. Though there is currently no evidence that the shorter treatment regimens will improve on adherence, reducing the treatment episode length will give patients more confidence that they can complete treatment, reduce the cost associated with accessing treatment and possibly the side effects associated with prolonged treatment periods.

## Figures and Tables

**Table 1:** Socio-demographic characteristics of the study participants with MDR-TB at Mulago National Referral Hospital January 2012–May 2016, N=227

Variable	Frequency, N	Percentage %
**Sex** *		
Female	89	39.4
Male	137	60.6
**Age group**		
0 – 14	5	2.2
15 – 24	62	27.3
25 – 34	74	32.6
35 – 44	53	23.4
45 – 54	26	11.5
>54	7	3.1
**Marital status** **		
Married	80	38.5
Separated	32	15.4
Single	87	41.8
Widowed	9	4.3
**Employment** ***		
No	76	34.9
Yes	142	65.1
**Residence**		
Urban	109	48.0
Rural	118	52.0

* 1 missing sex variable

** 19 missing marital status

*** 9 missing employment status
